# Fracture of Anterior Spinous Process of Ilium by Muscular Contraction

**Published:** 1871-04

**Authors:** 


Fracture of Anterior Spinous Process of Ilium by Muscular Contraction.—Drs. S. Joy and J. W. McWhinnie report (*Canada Med. Journal*, Sep., 1870) a remarkable case of this in a very muscular youth, æt. 17, who, in a foot-race, in turning at a given point, felt something snap in his right hip, walked a few steps and fell. "On examination distinct motion and crepitus could be felt by pressure over the process, also by placing the thumb over the origin of the Sartorius and rotating the thigh. The fracture extended into the notch below, but there was no great tendency to displacement, save when the leg was abducted, thus placing the Sartorius upon the stretch, the process, doubtless, being partially kept in place by the fibres of the tensor vaginæ femoris arising from this process on the one hand, and Poupart's ligament on the other, when tension was taken off the Sartorius. The patient was placed in bed with the thigh flexed and the shoulders raised, a bandage being applied to aid in steadying the fracture. It may be as well to state that this position and abduction of the right leg was maintained by bands attached to the posts of the bed. In two weeks the patient made a good recovery without displacement.—*American Jour. Med. Sciences.*

